# The *Drosophila* embryo as a *tabula rasa* for the epigenome

**DOI:** 10.12703/r/11-40

**Published:** 2022-12-23

**Authors:** Kami Ahmad, Steven Henikoff

**Affiliations:** 1 Division of Basic Sciences, Fred Hutchinson Cancer Center, 1100 Fairview Ave. N., P.O. Box 19024, Seattle, WA 98109-1024, USA; 2 Howard Hughes Medical Institute, 4000 Jones Bridge Road Chevy Chase, MD 20815-6789, USA

**Keywords:** Chromatin, Pioneer factors, Gene regulation

## Abstract

The control of gene expression in eukaryotes relies on how transcription factors and RNA polymerases manipulate the structure of chromatin. These interactions are especially important in development as gene expression programs change. Chromatin generally limits the accessibility of DNA, and thus exposing sequences at regulatory elements is critical for gene expression. However, it is challenging to understand how transcription factors manipulate chromatin structure and the sequence of regulatory events. The *Drosophila* embryo has provided a powerful setting to directly observe the establishment and elaboration of chromatin features and experimentally test the causality of transcriptional events that are shared among many metazoans. The large embryo is tractable by live imaging, and a variety of well-developed tools allow the manipulation of factors during early development. The early embryo develops as a syncytium with rapid nuclear divisions and no zygotic transcription, with largely featureless chromatin. Thus, studies in this system have revealed the progression of genome activation triggered by pioneer factors that initiate DNA exposure at regulatory elements and the establishment of chromatin domains, including heterochromatin, the nucleolus, and nuclear bodies. The *de novo* emergence of nuclear structures in the early embryo reveals features of chromatin dynamics that are likely to be central to transcriptional regulation in all cells.

## Introduction

Development in multicellular organisms relies on differential gene expression to direct cell proliferation and differentiation. The identification of conserved transcription factors in genetic screens for embryonic defects in *Drosophila* in the 1980s reformulated our concept of development^1,2^. Later studies in other organisms revealed that the genetic control of development is conserved across eukaryotes, with common regulatory principles. A key issue is how transcription factors interact with the chromatin packaging of the genome for differential gene expression, even though the genome in different cell types is identical. Just as exceptional features of *Drosophila* embryogenesis provided a productive setting for identifying developmental regulators, studies in early embryos are now unraveling long-standing issues of fundamental mechanisms of the chromatin control of transcription.

## The onset of chromatin organization in *Drosophila* embryos

The challenge in understanding transcriptional mechanisms is ordering molecular events and dependencies in living cells. Chromatin features have been correlated to gene expression and repression in many cell types, but it is more difficult to distinguish which features cause others and which actually regulate gene activity. Features of *Drosophila* embryogenesis are now being exploited to address this gap.

**Figure 1.**
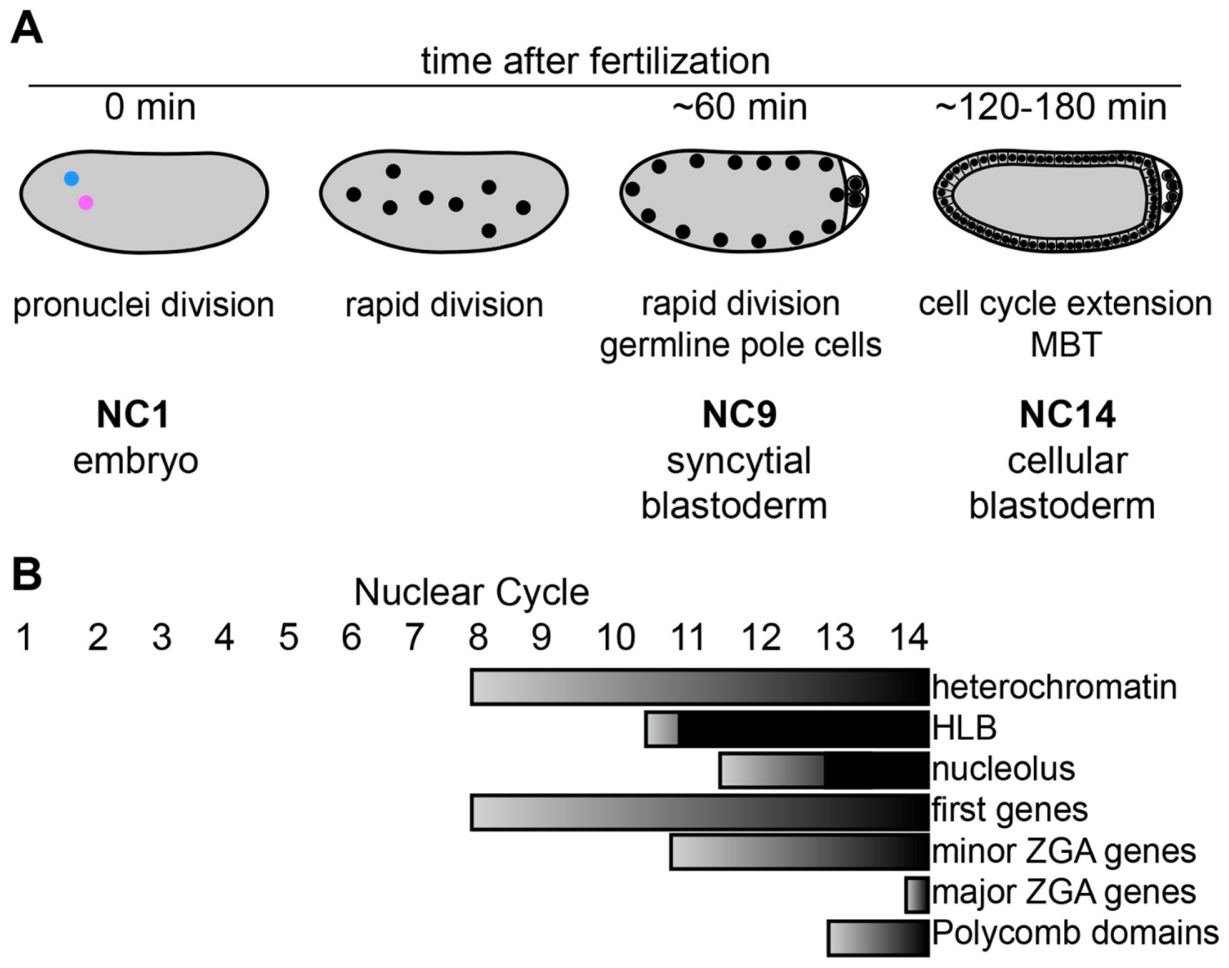
Chromatin events in early *Drosophila* embryos. (**A**) Outline of the early stages of *Drosophila* embryogenesis and proliferation. Nuclei are shown in black within the grey cytosol of the embryo. Stages are labeled as the number of nuclear cycles (nc) after fertilization. In nc1, protamines are removed from the male haploid pronucleus (blue) and repackaged with the H3.3 histone variant before DNA replication and mitosis. Fusion of the male and female pronuclei occurs in nc2. The first nuclear cycles up to nc13 occur in a syncytium with rapid progression through the S phase and mitosis without gap phases. (**B**) The timing of the appearance of chromatin features, including heterochromatin, Polycomb domains, and the histone locus bodies (HLBs), is shown. About 10 genes first activate in nc8, hundreds of genes activate in the minor zygotic genome activation (ZGA) around nc11, and thousands of genes activate in the major ZGA around nc14.

Like most animals, *Drosophila* females make a large egg that is loaded with proteins and untranslated mRNA. Fertilization by an incoming sperm triggers embryogenesis. A broad outline of chromatin changes in early embryogenesis is shown in Figure 1, where stages are labeled by the number of nuclear cycles (nc) since fertilization. The early stages of embryogenesis rely exclusively on maternally provided material, without any zygotic gene expression. The incoming sperm genome arrives packaged in protamines, which are removed and replaced with the H3.3 histone variant^3^. In its first hours, the embryo develops as a multinucleate syncytium with totipotent nuclei. Proliferative nuclear cycles occur synchronously and extremely fast, doubling nuclei every 10 minutes, so DNA replication, chromatin assembly, and mitosis occur at what must be close to maximal rates. These first rounds of nuclear replication and division use maternally supplied core histones and a variant histone H1 called bigH1^4^ to duplicate chromatin. The rapidity of these divisions requires nearly simultaneous replication of the entire genome, with replication origins scattered at random roughly every 10 kb^5^. The speed of early divisions means that syncytial nuclei largely lack any of the localized chromatin features that characterize epigenomes in other cell types, but distinctions appear as nuclear cycles slow down, zygotic transcription begins, and the nuclei are partitioned to form the cellular blastoderm. In this way, the transition from the syncytial to the cellular embryo provides a *tabula rasa* for observing the onset and order of transcriptional programs and epigenomic specialization.

Zygotic transcription of a few genes begins around nc8, reaching a notable level of transcription from about 100 sex determination, cellularization, and early patterning genes around nc11, a time referred to as the minor zygotic genome activation (minor ZGA)^6,7^. Widespread transcription of thousands of genes begins at nc14, referred to as the major ZGA, during the switch from maternal to zygotic control of morphogenesis. But how does zygotic transcription begin in the early embryo, and how does this alter the chromatin landscape?

Active promoters and enhancers in eukaryotes are characterized by a nucleosome-depleted region (NDR) where transcription factors bind. A small number of transcription-independent NDRs are detected in the nuclei of early syncytial embryos, probably due to intrinsic anti-nucleosomal sequence features^8^. At this time, developmentally regulated genes lack any nucleosome phasing around promoters or regulatory elements, but NDRs appear when transcription begins. At least three transcription factors have been implicated in driving zygotic transcriptional activation. The zinc-finger protein Zelda (Zld) is first translated beginning around nc8 from maternal mRNA and binds at thousands of sites^9–11^. These early sites include the promoters and enhancers of genes activated in the minor ZGA but also many regulatory elements of genes that will not be activated until the major ZGA. A second transcription factor implicated in early embryo transcription is the transcription factor GAGA factor (GAF), whose binding first appears in nc9^12^. A second GAGA motif-binding protein called CLAMP is involved in the activation of some major ZGA genes^13^. These key transcription factors also cooperate with spatially localized transcription factors to establish the body plan of the embryo (for examples, see 14–18).

## Pioneer factors… how do they work?

How do Zld and GAF bind chromatin in early nuclei? In many developmental systems, pioneer transcription factors are thought to convert inaccessible chromatin at binding sites into exposed DNA and thereby trigger new programs of gene expression^19^. For example, the mammalian pluripotency factors Sox2 and Oct4 bind DNA wrapped on the surface of a nucleosome and either shift its position or displace histones, thereby creating an NDR; exposure of this DNA then allows binding of secondary transcription factors. Thus, creating an NDR at binding sites is a key step for activating developmentally regulated enhancers and promoters, underlying the ability of these factors to reprogram cells from diverse types. These properties were first identified for the archetypal pioneer factor FoxA1, which initiates endoderm development^19^. Recent structural studies of pioneer factors docked onto nucleosomes *in vitro* have fleshed out factors that may bind specific DNA motifs even when wrapped around a histone octamer and how the binding of one factor may enhance the binding of a second^20^. However, the mechanism by which pioneer factors first bind sites *in vivo* has been more difficult to pin down. For example, a large fraction of binding sites for the FoxA1 factor depends on the cell type where it is expressed, implying that other bound transcription factors influence where FoxA1 can bind^21,22^. Close examination of FoxA1/2-binding sites in differentiating endoderm cells showed that these sites are partially accessible even before these factors are produced^23,24^. This makes it difficult to determine the relationship between site accessibility and pioneer factor activity, especially as the relevant cell stages in the early embryo cannot be easily observed.

Since the *Drosophila* embryo starts with a largely featureless epigenome, it provides insight into the chromatin sequence of pioneering. The early factors Zld, GAF, and CLAMP are pioneer factors in that each can bind nucleosomal DNA *in vitro*^13,25,26^. The creative use of mutations, engineered constructs, or microinjection into the syncytial embryo is deciphering the molecular activities of these pioneer factors (Box 1). Depletion of Zld, CLAMP, or GAF from early embryos has identified sets of binding sites where new accessibility depends exclusively on only one factor^9,12,13,18,27,28^ and other sets that require a combination of Zld and CLAMP or of Zld and GAF. Thus, at some sites, a single factor suffices to drive accessibility; at others, multiple transcription factors cooperate. This resembles the interdependency of pioneer factors in mammalian systems that allow for context-specific binding.

**Box 1. Systems to manipulate chromatin components in the early *Drosophila* embryo**

In the *Drosophila* embryo, the first 13 nuclear divisions occur synchronously in a large syncytium with a shared cytoplasm and are accessible for both observing and manipulating chromatin. A wide range of methods and techniques have been used in the studies discussed here. Embryos can be precisely staged by staining fixed material with DNA dyes, and detailed pictures of gene activation and the formation of nuclear bodies have been obtained using antibody staining and DNA *in situ* hybridization combined with high-resolution microscopy. Other well-developed methods use maternal genotypes that load the egg with fluorescently-labeled histones, chromatin proteins, or transcription factors for live imaging during embryogenesis. Fluorescent protein fusions with transcription factors and RNA polymerase II (RNAPII) subunits have been tracked in this way, and nascent transcripts have been detected with fluorescent MS2 coat protein (MCP) fusions that bind engineered hairpins in mRNAs^29^. The embryonic syncytium can be manually injected without adversely affecting development, and studies have injected mRNA for fluorescent protein fusions with DNA-binding proteins to image specific DNA sequences or fluorescently-labeled antibody fragments to image specific histone modifications.

Genetics and injection provide strategies to test function in the early embryo. Compounds that interfere with transcription, DNA replication, or chromatin remodeling can be injected. Since the early embryo relies entirely on maternally provided products until the beginning of the cellular blastoderm stage, many components can be altered by modifying the genotype of the mother. A range of genetic strategies are also available. For factors that are required only in the embryo, mutant mothers will produce eggs that lack a specific factor. In other cases, an interesting factor may also be important in other stages of development. Here, mitotic recombination schemes have been designed that produce homozygous mutant germline clones from heterozygous mothers, thereby giving eggs that lack the factor. Finally, for factors that are essential in the female germline, double-stranded RNA targeting a specific factor can be loaded into the egg, and this can accomplish knockdown in the embryo^30^. More sophisticated strategies are now applied in the embryo using inducible degradation components maternally loaded into the egg^31^, or optogenetic inactivation^25,32^ to trigger the elimination of factors during early embryogenesis. One particular method inspired by ‘anchors away’ technology in budding yeast uses the *Drosophila* Jabba protein to rapidly deplete GFP-labeled factors in the early embryo. Jabba normally localizes to lipid droplets in the early embryo, but a Jabba-anti-GFP nanobody fusion can sequester any target proteins outside of nuclei. Maternally loaded or injected mRNA encoding Jabba-anti-GFP nanobody has been used to acutely deplete histone methyltransferase proteins^33^, Zld, and Mxc^34^.

Live imaging has been used to follow the binding of Zld and transcription in the nuclei of early embryos. While earlier studies had shown that DNA replication initiates immediately after mitosis, and transcription slightly later, a recent preprint has detailed the link between replication and transcriptional initiation^34^. Zld accumulates in small foci in nuclei, and clusters of RNA polymerase II (RNAPII) and nascent gene transcripts appear adjacent to Zld foci^17,35^. These authors used an innovative sequestration method to rapidly deplete Zld from the embryo. This system uses an anti-GFP nanobody fused to the Jabba protein, which normally binds and sequesters target proteins in cytosolic lipid droplets in the embryo. Expression of this “JabbaTrap” fusion in embryos with a target GFP fusion protein thus sequesters the target away from chromatin^33^. Injection of JabbaTrap mRNA into embryos with a Zld-GFP fusion showed that Zld is required for the formation of RNAPII clusters. Intriguingly, blocking DNA replication with multiple different inhibitors greatly reduces the formation of Zld clusters and of RNAPII clusters. Clusters that do form are delayed and smaller. Thus, DNA replication appears to enhance the activity of Zld in early embryos.

New nucleosomes behind DNA replication forks are assembled with acetylated H3 and H4 histones, and so while these modifications are enriched in early embryonic nuclei, most other histone modifications associated with transcriptional regulation are absent^36^. However, when Zld binds, nucleosomes around these sites acquire histone H3K27 acetylation^8^. CBP is the main H3K27 acetyltransferase, and eliminating the *Drosophila* homolog Nejire (Nej) in early embryos blocks RNAPII clustering and transcription^34^. This argues for a sequence of events as follows: first, DNA replication enhances the binding of Zld and clustering of RNAPII on chromatin; second, Zld stimulates histone acetylation by Nej; and, third, acetylation promotes RNAPII elongation^34^. But why would DNA replication enhance the activity of a nucleosome-binding pioneer factor? Perhaps the binding of Zld is enhanced by cell cycle-coupled protein modifications of the factor. Alternatively, early models for chromatin regulation in development proposed that the chromatin dynamics during DNA replication may provide a “window of opportunity” to bind transcription factors and start new transcriptional programs^37^. Nucleosome density and positioning are transiently reduced behind replication forks^38^. For some transcription factors, disorganized nucleosome positioning may delay binding, but others (like GAF) rapidly bind newly replicated DNA and position nucleosomes. Evidence that most Zld and RNAPII signals in early *Drosophila* embryos depend on DNA replication supports the idea of fast binding behind the replication fork. Importantly, a small fraction of Zld shows delayed binding in blocked embryos, and this might be due to the nucleosomal-binding activity of Zld. While this division between replication-dependent and nucleosomal-binding modes of Zld binding may be more extreme in the fast cycles of the early embryo, DNA replication may promote new transcriptional programming more generally.

The fast nuclear division cycle of early embryos has also revealed factor-specific distinctions for mitotic inheritance. While many transcription factors dissociate as mitotic chromatin condenses, a long-standing question in mammalian systems has been whether some factors persist in maintaining stable patterns of gene expression in cell lineages^39^. Indeed, both properties appear in the early *Drosophila* embryo. Live imaging of Zld protein shows that the factor releases from chromatin as mitosis initiates^40^, and the chromatin accessibility of Zld-bound sites is reduced as nuclei divide^28^. In contrast, GAF binds both interphase and mitotic chromatin, and mitotic GAF-bound sites remain accessible^41^. This difference appears to result from the longer residence time of GAF on chromatin that allows persistent binding to mitotic chromosomes. Thus, GAF fits the definition of a mitotic bookmarking transcription factor and appears to be functionally important, as imaging nascent transcription of a target promoter has shown GAF-dependent mitotically heritable expression^41^.

There are additional transcription factors that activate genes in the major ZGA^42–44^. These can be understood in the framework that pioneering activity is a matter of degree, as transcription factors vary in affinity from site to site and cooperatively interact with other chromatin proteins^45^. However, one distinct class comprises about 2,000 promoters for housekeeping genes that are activated in the major ZGA but lack binding by any known pioneer factors. Instead, these promoters are enriched for the H2AV histone variant (the *Drosophila* ortholog of H2A.Z and H2A.X variants)^46^. H2AV is deposited at these promoters well before these genes are zygotically expressed by a conserved SWR1-type chromatin remodeler called Domino. While the elimination of Domino from the early embryo prevents H2AV accumulation and gene activation, chromatin accessibility is not altered. It remains to be determined whether these sites are targeted by pioneering core promoter factors or whether intrinsic nucleosome-positioning features of their DNA sequences make them accessible.

## Accretion of nuclear organization

The *de novo* establishment of nuclear organization in the early embryo includes the appearance of nuclear bodies and distinctive chromatin regions, including the histone locus body (HLB), the nucleolus, and heterochromatin. Observation of how these features appear reveals the steps in establishing phase-separated compartments within the nucleus. The HLB first appears in nc11 when the histone genes are transcribed from a gene array on chromosome 2, but DNA-binding factors — including the CLAMP pioneer factor and the NPAT homolog Mxc — first arrive at the histone genes one cycle earlier in nc10^47^. This nuclear body appears to be anchored by a DNA-bound seed, while processing factors may localize into the HLB through interactions with histone mRNA^48^. A similar sequence drives the formation of the nucleolus, where transcription of the rDNA genes from arrays on the X and Y chromosomes begins in nc11, and processing factors appear in nc13^49^ (Figure 1). These studies have led to a model where the early nucleus is supersaturated for nuclear body components, and factor binding followed by transcription triggers reliable formation of structured bodies.

Observations in the early embryo have also detailed the temporal relationship between histone modifications and gene regulation. Chromatin in the early embryo is globally hyperacetylated because of replication-coupled nucleosome assembly, but active modifications, such as H3K4 and H3K36 methylations, first appear after gene transcription is initiated in the major ZGA^36^. Markers of Polycomb gene silencing, such as H3K27 methylation and Polycomb complexes, first accumulate in nc14^8,50^ (although a conflicting report described localized H3K27 methylation at all cycles of the early embryo^51^). In *Drosophila*, regulatory elements called Polycomb Response Elements (PREs) are required for gene silencing, and PREs first become accessible in nc11^52^. Many PREs are binding sites for GAF, so the general mechanism of pioneering activity initiating zygotic gene expression also applies to the establishment of developmental gene silencing.

The establishment of heterochromatin in the embryo may operate by a different mechanism. In most cell types, repetitive transposons and short satellite repeats replicate very late in the S phase and become wrapped in nucleosomes marked by histone H3K9 methylation, comprising a repressive heterochromatic state. In the fast cycles of the early embryo, all sequences replicate at the same time, but repetitive sequence blocks begin to show heterochromatic properties at different times in later stages. Some satellite blocks become compacted in nc8 and gain H3K9 methylation in nc12, which then accumulates to high levels in successive cycles as interphases get longer^33^. More widespread heterochromatic features, including delayed replication and then HP1 binding, are apparent in nc13 and nc14^53,54^. Intriguingly, the embryonic establishment of heterochromatin on a specific satellite block depends on its maternal presence, suggesting that maternally-loaded RNAs from this satellite are required to establish heterochromatin in the embryo^55^. Similarly, maternal PIWI-interacting RNAs (piRNAs) may work on other repetitive sequences in the nucleus^56^. These dependencies may be distinct from the activities that maintain mature heterochromatin in later cell types.

The power of observation in the early embryo is now being applied to deciphering how the 3D organization of the nucleus is established. Chromatin in the nucleus displays a hierarchy of interactions, where loops between regulatory elements organize genes within topologically associated domains (TADs), which are further aggregated into inactive and active compartments^57^. This organization is thought to result from both cohesin binding that constrains the chromatin fiber and from non-specific associations between decondensed transcribed chromatin and inactive chromatin. As the early *Drosophila* embryo is transcriptionally silent, this stage provides a natural setting to track the acquisition of nuclear organization. Mapping of 3D organization in *Drosophila* embryos revealed that only a few boundaries are present in very early embryos, and these coincide with constitutive H2AZ-containing promoters^46^. Additional TADs and compartments appear in nc8 when Zld binds to chromatin^58,59^. This order of events demonstrates that factor binding alone can create domains and that transcription is not needed. Factor binding at TAD boundaries and at looping elements creates a framework of invariant cell type-independent domains in embryos, but only a subset of these interactions are productive in specific cell types^59–61^. Direct imaging of individual nuclei in the *Drosophila* embryo has further refined our understanding of nuclear organization. While contact maps have led to the impression of extensive 3D organization within nuclei, tracing chromatin paths^62–64^ or imaging the juxtaposition of transcribing genes^35^ shows that 3D organization in individual nuclei is very diverse, with structures detected by contact mapping being rare and transient. This places important limits on the functional significance of higher-order structures for gene expression, where 3D interactions appear to be a pre-condition for gene activation but do not determine it.

## Conclusions

Whereas the core questions in chromatin biology were posed many years ago, the intricacy of many eukaryotic systems has complexified the answers. The blank slate of chromatin in the early embryo has provided a simple setting for probing the mechanics of how transcription factors first interact with chromatin, how phase-separated bodies within the nucleus are first established, and how 3D nuclear organization comes to be. The *Drosophila* embryo has revealed that the binding of a small number of key DNA-binding proteins can trigger the organization of much of the nucleus, which successive gene activity elaborates. The sequence by which nuclear features emerge implies that they are consequences of “sticky” weak electrostatic interactions. These issues even apply to universally accepted concepts, such as how enhancers act on promoters to activate transcription. While textbooks describe that promoters and enhancers loop together in 3D space to form an activating structure, such contacts are rare and transient in direct imaging. How can transient contacts promote the slower process of transcriptional activation? One class of models suggests that an enhancer leaves a long-lasting mark on promoters, perhaps as histone modifications or stabilizing bound factors, and the persistence of these effects at promoters stimulates transcription^62^. More radical models propose that enhancers produce diffusible molecules that move across nuclear distance to activate promoters^65^. The ability to resolve the sequence of nuclear events in time and space in the early embryo holds promise in understanding these fundamental issues in gene regulation.

## References

[1] Zeitlinger J Stark A : Developmental gene regulation in the era of genomics. *Dev Biol.* 2010; 339(2): 230–9. 10.1016/j.ydbio.2009.12.039 20045679

[2] Nüsslein-Volhard C Wieschaus E : Mutations affecting segment number and polarity in *Drosophila*. *Nature.* 1980; 287(5785): 795–801. 10.1038/287795a0 6776413

[3] Loppin B Bonnefoy E Anselme C : The histone H3.3 chaperone HIRA is essential for chromatin assembly in the male pronucleus. *Nature.* 2005; 437(7063): 1386–90. 10.1038/nature04059 16251970

[4] Pérez-Montero S Carbonell A Morán T : The embryonic linker histone H1 variant of *Drosophila*, dBigH1, regulates zygotic genome activation. *Dev Cell.* 2013; 26(6): 578–90. 10.1016/j.devcel.2013.08.011 24055651

[5] Blumenthal AB Kriegstein HJ Hogness DS : The units of DNA replication in **Drosophila* melanogaster* chromosomes. *Cold Spring Harb Symp Quant Biol.* 1974; 38: 205–23. 10.1101/sqb.1974.038.01.024 4208784

[6] Kwasnieski JC Orr-Weaver TL Bartel DP : Early genome activation in *Drosophila* is extensive with an initial tendency for aborted transcripts and retained introns. *Genome Res.* 2019; 29(7): 1188–97. 10.1101/gr.242164.118 31235656 PMC6633261

[7] Lott SE Villalta JE Schroth GP : Noncanonical compensation of zygotic X transcription in early **Drosophila* melanogaster* development revealed through single-embryo RNA-seq. *PLoS Biol.* 2011; 9(2): e1000590. 10.1371/journal.pbio.1000590 21346796 PMC3035605

[8] Li XY Harrison MM Villalta JE : Establishment of regions of genomic activity during the *Drosophila* maternal to zygotic transition. *eLife.* 2014; 3: e03737. 10.7554/eLife.03737 25313869 PMC4358338

[9] Harrison MM Li XY Kaplan T : Zelda binding in the early *Drosophila melanogaster* embryo marks regions subsequently activated at the maternal-to-zygotic transition. *PLoS Genet.* 2011; 7(10): e1002266. 10.1371/journal.pgen.1002266 22028662 PMC3197655

[10] Nien CY Liang HL Butcher S : Temporal coordination of gene networks by Zelda in the early *Drosophila* embryo. *PLoS Genet.* 2011; 7(10): e1002339. 10.1371/journal.pgen.1002339 22028675 PMC3197689

[11] Larson ED Komori H Gibson TJ : Cell-type-specific chromatin occupancy by the pioneer factor Zelda drives key developmental transitions in *Drosophila*. *Nat Commun.* 2021; 12(1): 7153. 10.1038/s41467-021-27506-y 34887421 PMC8660810

[12] Gaskill MM Gibson TJ Larson ED : GAF is essential for zygotic genome activation and chromatin accessibility in the early *Drosophila* embryo. *eLife.* 2021; 10: e66668. 10.7554/eLife.66668 33720012 PMC8079149

[13] Duan J Rieder L Colonnetta MM : CLAMP and Zelda function together to promote *Drosophila* zygotic genome activation. *eLife.* 2021; 10: e69937. 10.7554/eLife.69937 34342574 PMC8367384

[14] Yamada S Whitney PH Huang SK : The *Drosophila* Pioneer Factor Zelda Modulates the Nuclear Microenvironment of a Dorsal Target Enhancer to Potentiate Transcriptional Output. *Curr Biol.* 2019; 29(8): 1387–1393.e5. 10.1016/j.cub.2019.03.019 30982648 PMC6702943

[15] Yáñez-Cuna JO Dinh HQ Kvon EZ : Uncovering *cis*-regulatory sequence requirements for context-specific transcription factor binding. *Genome Res.* 2012; 22(10): 2018–30. 10.1101/gr.132811.111 22534400 PMC3460196

[16] Xu Z Chen H Ling J : Impacts of the ubiquitous factor Zelda on Bicoid-dependent DNA binding and transcription in *Drosophila*. *Genes Dev.* 2014; 28(6): 608–21. 10.1101/gad.234534.113 24637116 PMC3967049

[17] Mir M Stadler MR Ortiz SA : Dynamic multifactor hubs interact transiently with sites of active transcription in *Drosophila* embryos. *eLife.* 2018; 7: e40497. 10.7554/eLife.40497 30589412 PMC6307861

[18] Sun Y Nien CY Chen K : Zelda overcomes the high intrinsic nucleosome barrier at enhancers during *Drosophila* zygotic genome activation. *Genome Res.* 2015; 25(11): 1703–14. 10.1101/gr.192542.115 26335633 PMC4617966

[19] Iwafuchi-Doi M Zaret KS : Cell fate control by pioneer transcription factors. *Development.* 2016; 143(11): 1833–7. 10.1242/dev.133900 27246709 PMC6514407

[20] Balsalobre A Drouin J : Pioneer factors as master regulators of the epigenome and cell fate. *Nat Rev Mol Cell Biol.* 2022; 23(7): 449–64. 10.1038/s41580-022-00464-z 35264768

[21] Lupien M Eeckhoute J Meyer CA : FoxA1 translates epigenetic signatures into enhancer-driven lineage-specific transcription. *Cell.* 2008; 132(6): 958–70. 10.1016/j.cell.2008.01.018 18358809 PMC2323438

[22] Larson ED Marsh AJ Harrison MM : Pioneering the developmental frontier. *Mol Cell.* 2021; 81(8): 1640–1650. 10.1016/j.molcel.2021.02.020 33689750 PMC8052302

[23] Meers MP Janssens DH Henikoff S : Pioneer Factor-Nucleosome Binding Events during Differentiation Are Motif Encoded. *Mol Cell.* 2019; 75(3): 562–575.e5. 10.1016/j.molcel.2019.05.025 31253573 PMC6697550

[24] Cernilogar FM Hasenöder S Wang Z : Pre-marked chromatin and transcription factor co-binding shape the pioneering activity of Foxa2. *Nucleic Acids Res.* 2019; 47(17): 9069–9086. 10.1093/nar/gkz627 31350899 PMC6753583

[25] McDaniel SL Gibson TJ Schulz KN : Continued Activity of the Pioneer Factor Zelda Is Required to Drive Zygotic Genome Activation. *Mol Cell.* 2019; 74(1): 185–195.e4. 10.1016/j.molcel.2019.01.014 30797686 PMC6544384

[26] Tsukiyama T Becker PB Wu C : ATP-dependent nucleosome disruption at a heat-shock promoter mediated by binding of GAGA transcription factor. *Nature.* 1994; 367(6463): 525–32. 10.1038/367525a0 8107823

[27] Schulz KN Bondra ER Moshe A : Zelda is differentially required for chromatin accessibility, transcription factor binding, and gene expression in the early *Drosophila* embryo. *Genome Res.* 2015; 25(11): 1715–26. 10.1101/gr.192682.115 26335634 PMC4617967

[28] Blythe SA Wieschaus EF : Establishment and maintenance of heritable chromatin structure during early *Drosophila* embryogenesis. *eLife.* 2016; 5: e20148. 10.7554/eLife.20148 27879204 PMC5156528

[29] Garcia HG Tikhonov M Lin A : Quantitative imaging of transcription in living *Drosophila* embryos links polymerase activity to patterning. *Curr Biol.* 2013; 23(21): 2140–5. 10.1016/j.cub.2013.08.054 24139738 PMC3828032

[30] Staller MV Yan D Randklev S : Depleting gene activities in early *Drosophila* embryos with the "maternal-Gal4-shRNA" system. *Genetics.* 2013; 193(1): 51–61. 10.1534/genetics.112.144915 23105012 PMC3527254

[31] Caussinus E Kanca O Affolter M : Fluorescent fusion protein knockout mediated by anti-GFP nanobody. *Nat Struct Mol Biol.* 2011; 19(1): 117–21. 10.1038/nsmb.2180 22157958

[32] Huang A Amourda C Zhang S : Decoding temporal interpretation of the morphogen Bicoid in the early *Drosophila* embryo. *eLife.* 2017; 6: e26258. 10.7554/eLife.26258 28691901 PMC5515579

[33] Seller CA Cho CY O'Farrell PH : Rapid embryonic cell cycles defer the establishment of heterochromatin by Eggless/SetDB1 in *Drosophila*. *Genes Dev.* 2019; 33(7–8): 403–417. 10.1101/gad.321646.118 30808658 PMC6446540

[34] Cho CY Kemp JP Jr Duronio RJ : Mandatory coupling of zygotic transcription to DNA replication in early *Drosophila* embryos. *bioRxiv.* 2022. 10.1101/2022.01.04.474810

[35] Huang SK Whitney PH Dutta S : Spatial organization of transcribing loci during early genome activation in *Drosophila*. *Curr Biol.* 2021; 31(22): 5102–5110.e5. 10.1016/j.cub.2021.09.027 34614388 PMC8612988

[36] Rudolph T Yonezawa M Lein S : Heterochromatin formation in *Drosophila* is initiated through active removal of H3K4 methylation by the LSD1 homolog SU(VAR)3-3. *Mol Cell.* 2007; 26(1): 103–15. 10.1016/j.molcel.2007.02.025 17434130

[37] Wolffe AP : Implications of DNA replication for eukaryotic gene expression. *J Cell Sci.* 1991; 99(Pt 2): 201–6. 10.1242/jcs.99.2.201 1885667

[38] Ramachandran S Henikoff S : Transcriptional Regulators Compete with Nucleosomes Post-replication. *Cell.* 2016; 165(3): 580–92. 10.1016/j.cell.2016.02.062 27062929 PMC4855302

[39] Zaidi SK Young DW Montecino MA : Mitotic bookmarking of genes: A novel dimension to epigenetic control. *Nat Rev Genet.* 2010; 11(8): 583–9. 10.1038/nrg2827 20628351 PMC3033599

[40] Dufourt J Trullo A Hunter J : Temporal control of gene expression by the pioneer factor Zelda through transient interactions in hubs. *Nat Commun.* 2018; 9(1): 5194. 10.1038/s41467-018-07613-z 30518940 PMC6281682

[41] Bellec M Dufourt J Hunt G : The control of transcriptional memory by stable mitotic bookmarking. *Nat Commun.* 2022; 13(1): 1176. 10.1038/s41467-022-28855-y 35246556 PMC8897465

[42] Koromila T Gao F Iwasaki Y : Odd-paired is a pioneer-like factor that coordinates with Zelda to control gene expression in embryos. *eLife.* 2020; 9: e59610. 10.7554/eLife.59610 32701060 PMC7417190

[43] Soluri IV Zumerling LM Payan Parra OA : Zygotic pioneer factor activity of Odd-paired/Zic is necessary for late function of the *Drosophila* segmentation network. *eLife.* 2020; 9: e53916. 10.7554/eLife.53916 32347792 PMC7190358

[44] Hannon CE Blythe SA Wieschaus EF : Concentration dependent chromatin states induced by the bicoid morphogen gradient. *eLife.* 2017; 6: e28275. 10.7554/eLife.28275 28891464 PMC5624782

[45] Hansen JL Loell KJ Cohen BA : A test of the pioneer factor hypothesis using ectopic liver gene activation. *eLife.* 2022; 11: e73358. 10.7554/eLife.73358 34984978 PMC8849321

[46] Ibarra-Morales D Rauer M Quarato P : Histone variant H2A.Z regulates zygotic genome activation. *Nat Commun.* 2021; 12(1): 7002. 10.1038/s41467-021-27125-7 34853314 PMC8636486

[47] White AE Burch BD Yang XC : *Drosophila* histone locus bodies form by hierarchical recruitment of components. *J Cell Biol.* 2011; 193(4): 677–94. 10.1083/jcb.201012077 21576393 PMC3166876

[48] Hur W Kemp JP Jr Tarzia M : CDK-Regulated Phase Separation Seeded by Histone Genes Ensures Precise Growth and Function of Histone Locus Bodies. *Dev Cell.* 2020; 54(3): 379–394.e6. 10.1016/j.devcel.2020.06.003 32579968 PMC7423771

[49] Falahati H Pelham-Webb B Blythe S : Nucleation by rRNA Dictates the Precision of Nucleolus Assembly. *Curr Biol.* 2016; 26(3): 277–85. 10.1016/j.cub.2015.11.065 26776729 PMC5866055

[50] Chen K Johnston J Shao W : A global change in RNA polymerase II pausing during the *Drosophila* midblastula transition. *eLife.* 2013; 2: e00861. 10.7554/eLife.00861 23951546 PMC3743134

[51] Zenk F Loeser E Schiavo R : Germ line-inherited H3K27me3 restricts enhancer function during maternal-to-zygotic transition. *Science.* 2017; 357(6347): 212–216. 10.1126/science.aam5339 28706074

[52] Alhaj Abed J Ghotbi E Ye P : *De novo* recruitment of Polycomb-group proteins in *Drosophila* embryos. *Development.* 2018; 145(23): dev165027. 10.1242/dev.165027 30389849 PMC6288389

[53] Shermoen AW McCleland ML O'Farrell PH : Developmental control of late replication and S phase length. *Curr Biol.* 2010; 20(23): 2067–77. 10.1016/j.cub.2010.10.021 21074439 PMC3108027

[54] Yuan K Shermoen AW O'Farrell PH : Illuminating DNA replication during *Drosophila* development using TALE-lights. *Curr Biol.* 2014; 24(4): R144–5. 10.1016/j.cub.2014.01.023 24556431 PMC3977024

[55] Yuan K O'Farrell PH : TALE-light imaging reveals maternally guided, H3K9me2/3-independent emergence of functional heterochromatin in *Drosophila* embryos. *Genes Dev.* 2016; 30(5): 579–93. 10.1101/gad.272237.115 26915820 PMC4782051

[56] Wei KHC Chan C Bachtrog D : Establishment of H3K9me3-dependent heterochromatin during embryogenesis in **Drosophila* miranda*. *eLife.* 2021; 10: e55612. 10.7554/eLife.55612 34128466 PMC8285105

[57] Rowley MJ Corces VG : Organizational principles of 3D genome architecture. *Nat Rev Genet.* 2018; 19(12): 789–800. 10.1038/s41576-018-0060-8 30367165 PMC6312108

[58] Hug CB Grimaldi AG Kruse K : Chromatin Architecture Emerges during Zygotic Genome Activation Independent of Transcription. *Cell.* 2017; 169(2): 216–228.e19. 10.1016/j.cell.2017.03.024 28388407

[59] Espinola SM Götz M Bellec M : *Cis*-regulatory chromatin loops arise before TADs and gene activation, and are independent of cell fate during early *Drosophila* development. *Nat Genet.* 2021; 53(4): 477–86. 10.1038/s41588-021-00816-z 33795867

[60] Ing-Simmons E Vaid R Bing XY : Independence of chromatin conformation and gene regulation during *Drosophila* dorsoventral patterning. *Nat Genet.* 2021; 53(4): 487–99. 10.1038/s41588-021-00799-x 33795866 PMC8035076

[61] Batut PJ Bing XY Sisco Z : Genome organization controls transcriptional dynamics during development. *Science.* 2022; 375(6580): 566–70. 10.1126/science.abi7178 35113722 PMC10368186

[62] Xiao JY Hafner A Boettiger AN : How subtle changes in 3D structure can create large changes in transcription. *eLife.* 2021; 10: e64320. 10.7554/eLife.64320 34240703 PMC8352591

[63] Mateo LJ Murphy SE Hafner A : Visualizing DNA folding and RNA in embryos at single-cell resolution. *Nature.* 2019; 568(7750): 49–54. 10.1038/s41586-019-1035-4 30886393 PMC6556380

[64] Cardozo Gizzi AM Cattoni DI Fiche JB : Microscopy-Based Chromosome Conformation Capture Enables Simultaneous Visualization of Genome Organization and Transcription in Intact Organisms. *Mol Cell.* 2019; 74(1): 212–222.e5. 10.1016/j.molcel.2019.01.011 30795893

[65] Karr JP Ferrie JJ Tjian R : The transcription factor activity gradient (TAG) model: Contemplating a contact-independent mechanism for enhancer-promoter communication. *Genes Dev.* 2022; 36(1–2): 7–16. 10.1101/gad.349160.121 34969825 PMC8763055

